# Evolving strategies for osteoporosis management in postmenopausal women: From tradition to innovation

**DOI:** 10.1097/MD.0000000000041605

**Published:** 2025-02-14

**Authors:** Anum Akbar, Amna Zaheer, Manahil Mansha Kharal, Aqsa Komel, Muhammad Hamza Khan, Areeba Ahsan, Achit Kumar Singh

**Affiliations:** a Department of Pediatrics, University of Nebraska Medical Center, Omaha, NE; b Liaquat National Hospital and Medical College, Karachi, Pakistan; c Islam Medical and Dental College, Sialkot, Pakistan; d Nishtar Medical University, Multan, Pakistan; e Karachi Medical and Dental College, Karachi, Pakistan; f Foundation University Medical College, Islamabad, Pakistan; g Kist Medical College and Teaching Hospital, Lalitpur, Nepal.

**Keywords:** bone health, Osteoboost, osteopenia, Osteoporosis, postmenopausal, T-score

## Abstract

Osteoporosis is a chronic condition primarily affecting postmenopausal women, significantly impacting their well-being and quality of life. Traditional treatment approaches include medications, vitamins, and exercise, but there is a growing interest in alternative therapies that enhance bone health. This review was conducted by searching multiple databases, including PubMed, Medline, and Google Scholar, for studies related to osteoporosis treatment. Articles focusing on both traditional therapies such as bisphosphonates, calcium, and vitamin D supplementation, and newer advancements like vibration therapy and bone-building devices such as Osteoboost were included. Traditional treatments, such as vitamin supplementation, exercise, and bisphosphonates, remain foundational in osteoporosis management, helping to maintain bone density and reduce fracture risks. Recent developments, including vibration therapy and Osteoboost, show promising results in bone regeneration without the use of medication. While traditional therapies continue to play an essential role, advancements like vibration therapy present novel alternatives for managing osteoporosis. Further research is necessary to optimize these approaches, ensuring they maximize benefits while minimizing risks, ultimately improving patient outcomes and quality of life.

## 1. Introduction

Osteoporosis is a metabolic bone disease that results from decreased bone mineral density and bone mass. The net effect is increased structural risk of bone fractures in adults.^[1,2]^ Osteoporosis is sometimes referred to as a silent disease because bone loss occurs without any signs or symptoms until fractures occur. This is even seen with two-thirds of vertebral fractures where the underlying cause was osteoporosis.^[3]^ Osteoporosis is a common disease, affecting one in 3 women and one in 5 men over the age of 50 worldwide.^[4]^ A 2021 meta-analysis study reported the prevalence of osteoporosis in the world to be 18.3 (95% CI: 16.2–20.7), with a 23.1 (95% CI: 19.8–26.9) prevalence amongst women and 11.7 (95% CI: 9.6–14.1) prevalence amongst men.^[5]^ Looking at the gender disparity of osteoporosis between men and women, a study found that women aged 50 and above did have 4 times higher rate of osteoporosis and 2 times higher rate of osteopenia. The study also concluded that women tend to start having fractures 5 to 10 years earlier than men.^[6]^

Overall, the prevalence of osteoporosis in the world is very high, more so in Africa and Europe.^[5]^ According to a report by World Health Organization (WHO), osteoporosis affects approximately 200 million women worldwide, with 30% of white Caucasian postmenopausal females in USA suffering from osteoporosis.^[4,7]^

A study exploring the global burden of osteoporosis found that in the year 2019, 5 countries had the highest disease burden of disability-adjusted life-years number in low bone mineral density related fractures. These were India, China, USA, Japan, and Germany. The respective percentages of the global burden of disability-adjusted life-years and deaths associated with low bone mineral density and its related fractures in these countries were 25.59%, 18.75%, 8.35%, 3.29%, and 3.04%.^[8]^

The standard measure for the diagnosis of osteoporosis and the assessment of fracture risk is bone mineral density. The dual-energy X-ray absorptiometry scan measures bone mineral density and generates a T-score, and the WHO has defined osteoporosis based on dual-energy X-ray absorptiometry T-scores. According to the WHO criteria, normal bone density is a T-score within 1 SD (+1 or −1) of the young adult mean bone density. A T-score of 1 to 2.5 SD below the young adult mean bone density (−1 to −2.5 SD) indicates low bone mass, and the presence of osteoporosis is indicated by a T-score of 2.5 SD or more below the young adult mean (more than −2.5 SD).^[9]^

Osteoporosis has a significant negative impact on quality of life due to a substantially increased risk for fractures. One study demonstrated that patients with osteoporotic fractures had lower health-related quality of life as well as overall lower health status.^[10]^ Fractures are also associated with an increase in premature mortality, disability, and financial burden.^[11,12]^ Many environmental factors may increase or decrease risks for osteoporotic fractures. For example, in postmenopausal women, smoking is an independent risk factor whereas physical activity is a protective factor for bone mass retention.^[13]^ Osteoporosis has a profound impact on both health and economics, primarily through fragility fractures that commonly affect the hip, spine, and wrist. These fractures lead to substantial morbidity, with hip fractures often resulting in high mortality rates – about 31,000 excess deaths annually in the U.S. – and long-term disability, as 50% of those who could walk independently before a hip fracture lose that ability afterward. Quality of life is significantly diminished due to chronic pain, reduced mobility, and psychological effects such as depression and social isolation. The economic burden is equally severe; in 1990, global direct and indirect costs of hip fractures were estimated at $34.8 billion, projected to rise to $131.5 billion by 2050. In the U.S. alone, osteoporosis-related fractures cost $19 billion annually, while in the UK, healthcare costs for fragility fractures amount to £1.7 billion each year, with hip fractures accounting for a third of this. As the global population ages, the number of fractures is expected to increase dramatically, with hip fractures projected to rise from 1.66 million in 1990 to 6.26 million by 2050. This underscores the urgent need for preventive strategies to reduce both the health and economic burden of osteoporosis.^[14]^ In postmenopausal white or Caucasian women, additional risk factors include low calcium intake, heavy alcohol consumption, and use of long half-life psychotropic drugs. Protective factors include ingesting vitamin D and its metabolites, 2 ppm or more of fluoride in drinking water, breastfeeding, and using thiazide diuretics or progesterone.^[15]^ Bone strength relies on various factors such as bone structure, thickness of the outer layer (cortical bone), presence of pores within the bone, internal structure of trabecular bone, and inherent qualities of the bone tissue. Dual-energy X-ray absorptiometry (DXA) is commonly used to indirectly gauge bone strength by measuring bone mineral density (BMD).^[16]^ The Fracture Risk Assessment Tool incorporates various clinical risk factors alongside a patient’s BMD T-scores to assess the likelihood of future fractures.^[17]^ These factors encompass demographic details such as age, gender, ethnicity, height, weight, and body mass index, as well as medical history elements like prior fragility fractures, family history of hip fractures, use of oral glucocorticoids, presence of rheumatoid arthritis or other conditions leading to secondary osteoporosis, current smoking status, and alcohol consumption.^[17]^

In 1999, the Bone Health and Osteoporosis Foundation (formerly known as the National Osteoporosis Foundation) first published the Clinician’s Guide to serve as an accurate information source about the prevention and treatment of osteoporosis. It provides brief guidelines on prevention, risk assessment, diagnosis, and management, along with fracture risk thresholds for the need for pharmaceutical intervention. Though improvements have been made in diagnostics and treatments, postmenopausal women with osteoporosis show a worse quality of life.^[18]^ This warrants the need for effective and preventive treatment strategies. Current treatment options include both pharmacological and non-pharmacological options to prevent fractures, but these treatments are not suitable for all individuals. Calcium and vitamin D supplementation may improve bone density, but patients taking levothyroxine for thyroid disease decreased absorption of these supplements.^[19]^ Combined hormone replacement therapy as a treatment for post-menopausal women still remains controversial due to the many side effects.^[20]^ Bisphosphonates (BPs) have been shown to improve bone mineral density in postmenopausal women with osteoporosis and are widely prescribed; however, BPs also present with many side effects such as infusion reactions, hypocalcemia, myalgias, arthralgias, and various gastrointestinal adverse effects.^[21]^

Two recent announcements were made by the Food and Drug Administration (FDA) about the management of a single shift in treatment strategies for osteoporosis. First, the FDA announced a new requirement for a “Boxed Warning” for increased risk of severe hypocalcemia in patients with advanced chronic kidney disease taking the osteoporosis medicine Prolia (denosumab). Second, the FDA announced the approval of the device “Osteoboost” – a wearable belt that delivers calibrated vibration to the hips and lumbar spine and is presumed to prevent decreases in bone density in postmenopausal women.^[22,23]^

The objectives of this review are to critically review the current treatment options available for osteoporosis and compare traditional (<2019) treatment options with recent (≥2019) treatment options.

## 2. Method

A comprehensive literature search was conducted using PubMed and Google Scholar to explore evolving strategies for osteoporosis management in postmenopausal women. Search terms included “osteoporosis,” “postmenopausal women,” “bone density,” “fracture prevention,” “treatment,” “management strategies,” “traditional therapies,” “innovative therapies,” and “clinical outcomes,” employing MeSH terms and Boolean operators. The search covered in vitro, in vivo, and human studies without time restrictions. Titles and abstracts were screened to apply inclusion criteria (studies on osteoporosis management in postmenopausal women, both traditional and innovative strategies, and English language) and exclusion criteria (irrelevant topics, editorials, commentary, etc.). Selected articles were reviewed, and additional relevant studies were identified from references. Data were extracted and synthesized to provide an overview of traditional and innovative management strategies, including the application of advanced technologies in osteoporosis care.

## 3. Pathophysiology of osteoporosis

At cellular levels, sex steroid deficiency and aging lead to an increase in bone resorption and a decrease in bone formation by the osteoclasts and the osteoblasts, respectively.^[24,25]^ Certain pro-inflammatory cytokines that regulate osteoclast differentiation, including TNF-α, IL1, IL-6, and receptor activators of the activator of nuclear factor (NF) Kappa-B ligand receptor (RANKL)/Osteoprotegerin/RANK system, are associated with a deficiency of estrogen in the bone microenvironment.^[26]^ The peptide RANKL on the osteocytes interacts with the RANK receptor present on the osteoclast precursors. This interaction results in differentiated multinucleated osteoclasts. The macrophage colony-stimulating factor present in the osteoblast also plays a role in the osteoclast precursor differentiation.^[27,28]^ Furthermore, chemokines produced by the osteoblasts help in osteoclast precursor recruitment and in the degradation of un-mineralized osteoid by the matrix metalloproteinases.^[28]^ Hydrogen ions and lysosomal enzymes secreted by the osteoclasts lead to acidification and proteolysis, which removes previous bone.^[29]^ However, the ligand Osteoprotegerin can block the RANK-RANKL interaction and reduce bone resorption. Thus, inhibiting the osteoclasts to differentiate and enhance their apoptosis. In age-related osteoporosis, altered lineage distribution of Bone Marrow Stromal Cells (BMSCs), with the increased differentiation of the adipocyte lineage, leads to a buildup of Bone Marrow Adipose Tissue, which jeopardizes the differentiation of osteoblasts and bone formation.^[30]^

## 4. Traditional treatment for osteoporosis

### 4.1. Role of vitamins

Maintaining optimal bone health requires adequate calcium and vitamin D intake.^[31]^ High-calcium foods, supplements, and exposure to sunlight are recommended, especially for higher-risk groups such as older individuals and those on glucocorticoid therapy. While vitamin D supplementation at 800 IU/day and calcium at 1000 to 1200 mg/day is generally advised for postmenopausal women, individualized dosages may be necessary.^[32]^ A study has shown that in women aged 65 years or older, dietary supplementation containing calcium and vitamin D showed a moderate decrease in bone loss.^[33]^ However, caution is urged regarding widespread calcium supplement use in postmenopausal women with dyslipidemia due to a potential for an increase in serum total cholesterol concentration and carotid intima thickness.^[34]^

A study revealed a positive correlation between water-soluble vitamins and BMD while demonstrating a negative association between fat-soluble vitamins and BMD.^[35]^

Other nutrients, such as magnesium, potassium, and polyunsaturated fatty acids, also play a role in bone health, emphasizing the importance of a well-balanced diet.^[36]^ Supplementation with B vitamins, omega-3 fatty acids, soy isoflavones, and dehydroepiandrosterone are emerging interventions that may offer additional avenues for bone health improvement and may help reduce the risk of osteoporosis when consumed as part of a well-rounded diet.^[37,38]^ Regular follow-up and individualized dietary plans contribute to comprehensive bone health management.

### 4.2. Role of lifestyle modifications

High-intensity resistance training significantly boosts BMD at the lumbar spine and femoral neck, while regular walking benefits femoral neck BMD. However, low to moderate-intensity physical activity is considered more effective than high-intensity exercises for optimizing BMD.^[39]^ Previous study has shown that regular exercise decreases the risk of osteoporosis in postmenopausal women.^[40]^ Fall prevention measures, such as exercises to improve balance and strength, home modifications, and the use of aids like grab bars, are crucial in reducing fracture risks associated with osteoporosis. Healthcare providers should promote these lifestyle changes to support bone health and prevent complications. These findings are supported by various sources, including studies by Martyn and Carroll, guidelines emphasizing fall prevention, and recommendations for postmenopausal women.^[41–44]^

### 4.3. Medications

BPs inhibit bone resorption, while Denosumab neutralizes RANKL, and Teriparatide stimulates bone remodeling. Estrogen agonists, calcitonin, and hormone therapy may also play a role in the medical management of osteoporosis. Understanding medication mechanisms and efficacy aids in personalized treatment approaches.

#### 4.3.1. Bisphosphonates

For postmenopausal women at a high risk of fractures, initial treatment with BPs (alendronate, risedronate, zoledronic acid, and ibandronate) or denosumab is recommended.^[45]^ BPs bind to bone minerals and are internalized by mature osteoclasts at sites of bone resorption.^[46]^ Amino-bisphosphonates contain nitrogen, which impairs osteoclast function by inhibiting farnesyl pyrophosphate synthase (FPPS), thereby preventing the prenylation of small GTPase proteins. GTPase disruption leads to disruptions in cytoskeletal organization, loss of the ruffled membrane border, and altered vesicular trafficking,^[47–50]^ which ultimately accelerates osteoclast apoptosis and prevents bone resorption.^[48]^

BPs can remain bound to bone minerals for many years; those with higher binding affinities (zoledronic acid > alendronate > ibandronate > risedronate) have longer skeletal residency.^[51]^ As a result, even after discontinuation, bound BPs continue to exert residual pharmacological action for an extended period, which is in contrast to other anti-resorptive therapies that quickly lose activity after discontinuation (e.g., denosumab, estrogen, raloxifene, and calcitonin).^[51–55]^ The primary adverse effects linked with BPs include renal toxicity, acute-phase reactions, gastrointestinal (GI) issues, and osteonecrosis of the jaw.^[56]^

#### 4.3.2. Denosumab

Denosumab is a fully human monoclonal antibody that functions as a potent inhibitor of bone resorption by specifically targeting the RANKL. Due to denosumab’s high affinity and specificity for RANKL, denosumab impedes osteoclast differentiation, function, and survival.^[57,58]^ In contrast to BPs, denosumab effectively suppresses osteoclast activity across all developmental stages (including prefusion, multinucleated, and resorbing mature osteoclasts), resulting in improved accessibility to bone remodeling compartments in cortical bone compared to BPs.^[46,59]^ The most common side effects of denosumab include rash, pruritis, and urticaria.^[60]^ Recently FDA approved a warning for denosumab causing severe hypocalcemia in patients with advanced chronic kidney disease. Patients often develop severe hypocalcemia 2 to 10 weeks after each denosumab injection, with the highest risk between Weeks 2 to 5. This posed complications, including arrhythmias, for some, particularly those on dialysis, exhibiting signs such as confusion, seizures, facial twitching, and muscle weakness.^[22]^

#### 4.3.3. Parathyroid hormone (PTH) analogues

The medication is recommended for glucocorticoid-induced osteoporosis and severe osteoporosis in postmenopausal women and men.^[61]^ Evident from the randomized controlled trials over the years, it’s proven that Teriparatide (Forteo), a recombinant PTH, can reduce the risk of vertebral and nonvertebral fractures faster and to a greater extent than antiresorptive treatments that decrease both bone formation and bone resorption.^[62,63]^ It can also increase BMD at the spine and hip, both important sites for fracture prevention, and improves bone quality and architecture by increasing trabecular and cortical bone volume and thickness.^[64,65]^ However, owing to the potential risk of osteosarcoma, a rare type of bone cancer, the drug has a limited duration of use. Additionally, teriparatide is not suitable for patients with certain medical conditions, such as hyperparathyroidism, kidney stones, or Paget’s disease.^[66]^

#### 4.3.4. Estrogen agonist

The absence of estrogen following menopause accelerates bone loss and heightens the likelihood of fractures, and a study has shown a dose-dependent effect of estrogen on bone turnover and BMD changes. Menopausal hormone therapy (MHT) in postmenopausal women effectively prevents bone loss and deterioration of bone structure, thereby reducing fracture risk by 20% to 40% across all bone sites.^[67,68]^ Interestingly, MHT is the only type of treatment that has shown efficacy in preventing osteoporosis, even in low-risk postmenopausal women.^[69]^ Research indicates that continuous use of Hormone Replacement Therapy (HRT) is necessary for it to effectively prevent fractures. However, long-term usage of combined HRT is associated with an increased risk of breast cancer diagnosis.^[19]^ Furthermore, estrogen therapy, regardless of administration method (oral or transdermal), has a favorable impact on bone health. Nevertheless, research indicates that postmenopausal women may need more than 7 years of estrogen replacement therapy to effectively maintain bone mineral density over the long term.^[70]^ Like combined HRT, long-term estrogen replacement therapy also increases the risk of breast cancer. Study has shown that raloxifene, a selective estrogen receptor modulator, is effective (though less effective than estrogen) in prevention and treatment of postmenopausal osteoporosis.^[71]^ it’s important to exercise caution when using raloxifene due to its association with an elevated risk of thromboembolism.^[72]^

#### 4.3.5. Calcitonin

Calcitonin is a peptide composed of 32 amino acids which binds to osteoclasts and inhibits bone resorption.^[73]^ Calcitonin serves 3 primary functions: facilitating calcium absorption into bones, inhibiting calcium reabsorption in the kidneys, and inhibiting calcium reabsorption in the small intestine. Consequently, in osteoporosis treatment, calcitonin operates by promoting calcium storage within bones.^[74]^ However, the primary issue with calcitonin treatment is that more effective drugs, such as BPs, are readily accessible for preventing bone loss and reducing fracture risk.^[32]^

## 5. Recent advancements in treating osteoporosis

### 5.1. Romosozumab

Romosozumab, a sclerostin inhibitor, is a humanized monoclonal antibody used to treat osteoporosis in postmenopausal women at high fracture risk. Romosozumab promotes osteogenesis and partially inhibits bone resorption by facilitating the Wnt/β-catenin pathway.^[75–77]^ Romosozumab’s action involves blocking sclerostin, allowing Wnt/β-catenin signaling to stimulate osteoblast development and bone growth.^[78,79]^ Romosozumab significantly reduces the risk of vertebral, nonvertebral, and clinical fractures in postmenopausal women with osteoporosis. In one clinical trial, romosozumab reduced the risk of vertebral fractures by 73% compared with placebo and by 48% compared with alendronate (PTH analog). Additionally, Romosozumab reduced the risk of nonvertebral fractures by 36% compared with placebo and by 27% compared with alendronate.^[80]^ Phase II research demonstrated its safety and efficacy, showing a substantial increase in lumbar spine bone mineral density by 11.3% with a monthly 210 mg dose.^[21]^ The FRAME trial confirmed romosozumab’s ability to protect postmenopausal women from vertebral fractures, with a significantly lower incidence compared to a placebo.^[75]^ Common side effects include headaches, muscular spasms, and arthralgia, with injection-site responses more prevalent than a placebo.^[28]^ A prominent consideration with romosozumab revolves around its notable cardiovascular risk profile. In the ARCH trial, it was revealed that where romosozumab reduces the risk of fractures, it increases the risk of serious cardiovascular adverse events by 30% compared with alendronate. This difference was mainly driven by a higher rate of myocardial infarction in patients treated with romosozumab.^[81]^

### 5.2. Vibration therapy

Vibration therapy uses high-frequency, low-magnitude vibrations to stimulate muscles and bones. There are primarily 2 types of vibrating devices: whole-body vibration devices and single-muscle vibration devices. Space agencies have used whole-body vibration therapy for astronauts returning from space missions to improve BDM.^[82]^ Both animal and human research indicate that high-frequency, low-magnitude vibration therapy enhances bone strength by promoting bone formation and reducing bone resorption.^[83]^ The mechanism of action includes sending anabolic signals to bone, muscles, and tendons, improving blood circulation, and differentiating stem cells into osteoblasts. It also reduces the formation of excessive osteoclasts by gap junction communication in osteocytes.^[82]^ Vibration therapy reduces pain and fatigue, enhances muscle strength and flexibility, aids in rehabilitation, and boosts bone density. However, they lack a standardized protocol for therapy.^[84]^

Osteoboost is a novel wearable device designed to enhance bone strength and density and deliver focused vibrations to the lumbar spine and hips. It has an incorporated motor designed to transmit low-amplitude, high-frequency (20–40 Hz) vibrations to the spine and hips. The device consists of a vibration pack mounted inside a nylon bag, which delivers mechanical stimulation to bones, mimicking the effects of physical activity. This technology helps improve bone density and strength, particularly in individuals with osteoporosis, reducing the risk of fractures.^[85]^ A recent clinical trial investigating the effect of osteoboost in 126 post-menopausal women with low bone mass who were not taking bone-active medications showed a significant difference in loss of vertebral bone strength for women in the 50 to 60-year-old category. In patients meeting the compliance criteria of 3x/week, there was a 5 times greater loss in the sham group compared to the active group. This study indicates that osteoboost treatment has positive effects in preserving volumetric bone density and vertebral bone strength in postmenopausal women with low bone mass.^[85]^ Osteoboost Belt has undergone a review by the Center for Devices and Radiological Health (CDRH) of FDA and has been classified as a prescription device. It is indicated for post-menopausal women suffering from ostopenia (BMD T-score of −1.0 to −2.49) of the lumbar vertebrae or total hip to reduce volumetric bone density decline as well as a decline in bone strength.^[86]^

Osteoboost presents a novel approach to treating low bone density in postmenopausal women, utilizing vibration to stimulate bone formation and prevent fractures in the spine and hips. This technology has demonstrated both clinical efficacy and safety in pivotal trials. With the convenience of being medication-free and FDA-cleared, Osteoboost offers promise. However, its availability is currently limited, and individual responses may vary. Determining the cost-effectiveness and optimal usage duration required to maintain this therapy’s benefits is also essential. Patients intrigued by Osteoboost should discuss its potential benefits and risks with their healthcare providers. Adopting a healthy lifestyle and ensuring adequate intake of calcium and vitamin D are recommended to complement this treatment approach.^[87]^

## 6. Traditional vs recent treatment options

Table 1 contrasts the effectiveness and adverse effects of traditional treatment approaches (year < 2019) with recent treatment options (year ≥ 2019) for osteoporosis in postmenopausal women.^[88–95]^

**Table 1:** contrasts the effectiveness and adverse effects of traditional treatment approaches (year < 2019) with recent treatment options (year ≥ 2019) for osteoporosis in postmenopausal women.^[88–92]^

	Teriparatide	Denosumab	Bazedoxifene	Romosozumab	Osteoboost
Year FDA approved	2003	2010	2013	2019	2024
Mechanism of action	It stimulates osteoblasts, and when given intermittently, it increases calcium absorption and retention, thereby improving bone density and reducing the likelihood of fractures in individuals with osteoporosis.^[93]^	It inhibits RANKL, which prevents osteoclast development and decreases bone resorption, leading to higher bone density and a reduced risk of fractures in osteoporosis.^[94]^	It inhibits bone resorption via estrogen receptor activation in bone tissue, which increases bone density and lowers the risk of fractures in postmenopausal osteoporosis.^[95]^	It suppresses sclerostin, promoting osteoblast activity and new bone formation while decreasing bone resorption. This combined action boosts bone mineral density and lowers the risk of fractures in osteoporosis.^[92]^	It provides high-frequency, low-magnitude vibrations that stimulate bone formation, reduce bone resorption, and improve blood circulation. It enhances osteoblast activity, limits osteoclast development via osteocyte signaling, and strengthens muscles, leading to improved bone density and fracture prevention.^[85]^
Efficacy	56%	72%	20–40%	73%	5–10%
FDA box warning	Though FDA has removed the warning regarding Osteosarcoma, it still recommends to avoid in patients with an increased risk of osteosarcoma^[96]^	Increased risk of severe hypocalcemia in patients with advanced chronic kidney disease (CKD)^[22]^	Endometrial cancer, cardiovascular disorders and dementia^[97]^	Potential risk of myocardial infarction (MI), stroke, cardiovascular death^[98]^	NA

While there are several treatment options available for osteoporosis, almost all of them have FDA box warnings for serious consequences (Table 1).^[96–98]^ Considering this, the newer vibration device, Osteoboost, for the management of osteoporosis may be a safe and alternate option.

## 7. Conclusion

In conclusion, traditional treatment modalities such as PTH analogs, hormone therapy, and denosumab have demonstrated promising outcomes in managing osteoporosis. However, each option carries distinct side effects, and the FDA box warning, including the recent FDA warning regarding denosumab-induced hypocalcemia, underscores the need for alternative approaches. Osteoboost, a non-pharmacological intervention utilizing vibration therapy, presents itself as a promising alternative with potentially fewer side effects. Further investigation through long-term studies is imperative to compare the efficacy and cost-effectiveness of Osteoboost against available monoclonal antibodies and medications for osteoporosis.

## Author contributions

**Conceptualization:** Anum Akbar.

**Project administration:** Anum Akbar.

**Supervision:** Anum Akbar.

**Visualization:** Muhammad Hamza Khan.

**Writing – original draft:** Amna Zaheer, Manahil Mansha Kharal, Aqsa Komel, Muhammad Hamza Khan, Areeba Ahsan, Achit Kumar Singh.

**Writing – review & editing:** Anum Akbar.
